# Pancreatic Pseudocyst Masquerading as Right Atrial Thrombus: An Unusual Intracardiac Extension

**DOI:** 10.7759/cureus.114861

**Published:** 2026-08-20

**Authors:** Hariprasad Ramanathan, Rathod Subraj, Gurumurthy Balasubramanian

**Affiliations:** 1 Radiology, Jawaharlal Institute of Post Graduate Medical Education and Research, Pondicherry, IND

**Keywords:** acute pancreatitis complications, caval wall defect, computed tomography scan, inferior vena cava (ivc), intracardiac extension, pigtail catheter drainage, pseudocyst of pancreas, right atrial cardiac mass, thrombus mimic, transabdominal ultrasound

## Abstract

Pancreatic pseudocysts are encapsulated collections of enzyme-rich pancreatic secretions that may extend beyond the peripancreatic region. Extension into the mediastinum and heart is uncommon and may mimic other thoracic or intracardiac pathologies.

A 36-year-old man with chronic alcohol use presented with abdominal pain, fever, dyspnoea and palpitations on a background of recurrent alcohol-related pancreatitis. Contrast-enhanced CT demonstrated acute-on-chronic pancreatitis with multiple pancreatic pseudocysts and a right atrial filling defect initially interpreted as thrombus. Focused ultrasound, including a subxiphoid four-chamber view, demonstrated an avascular anechoic lesion within the right atrium, continuous inferiorly with an abdominal pseudocyst along the course of the inferior vena cava. A retrospective review of the CT confirmed this continuity. The findings indicated cranial extension of the collection along the periadventitial plane of the retrohepatic cava, with full-thickness breach of the caval wall at the caval-atrial junction and herniation of the collection into the lumen enclosed by its own fibrous capsule, without communication between the cyst cavity and the circulating blood. Cyst fluid was markedly amylase- and lipase-rich. Ultrasound-guided percutaneous drainage of the abdominal component produced regression of both components without embolic or hemorrhagic complications.

Misinterpretation carries substantial consequence. A right atrial filling defect attributed to thrombus commits the patient to therapeutic anticoagulation at a time when acute pancreatitis independently predisposes to retroperitoneal and peripancreatic hemorrhage, arterial pseudoaneurysm formation and vascular erosion, and may additionally prompt consideration of thrombolysis or cardiac surgical thrombectomy. Correct characterisation therefore alters management directly and immediately.

## Introduction

Acute pancreatitis is a common inflammatory condition with a wide spectrum of local and systemic complications [1]. Pancreatic pseudocysts represent encapsulated collections of pancreatic secretions surrounded by a fibrous wall, typically developing several weeks after an acute insult or in the setting of chronic pancreatitis with recurrent exacerbations [2]. These collections may extend beyond the peripancreatic region by tracking along fascial planes due to enzymatic activity and increased intracystic pressure [3].

Ectopic locations of pancreatic pseudocysts have been described, including the mediastinum, pelvis, spleen, liver, and perirenal space [3,4]. However, extension into the cardiovascular system is extremely uncommon. Intracardiac involvement, particularly within the right atrium, is exceedingly rare and may mimic other intracardiac pathologies such as thrombus [5].

Misinterpretation as thrombus commits the patient to therapeutic anticoagulation at a time when acute pancreatitis independently predisposes to retroperitoneal hemorrhage and vascular erosion, and may prompt consideration of thrombolysis or cardiac surgical thrombectomy.

We report a rare case of pancreatic pseudocyst extending along the inferior vena cava and herniating into the right atrium, highlighting the role of multimodality imaging in diagnosis and management.

## Case presentation

A 36-year-old male with a history of chronic alcohol use presented with abdominal pain, fever, dyspnea, and palpitations. He had experienced four prior episodes of symptomatic pancreatitis over the preceding three years, the most recent being three months before admission, establishing this as an acute exacerbation on a background of chronic alcohol-related pancreatitis. The abdominal pain was intermittent, pricking in nature, and aggravated after meals. Initial laboratory investigations were consistent with acute pancreatitis and a significant systemic inflammatory response. The patient's hematological and biochemical parameters on presentation and following drainage are summarized in Table 1.

**Table 1:** Laboratory parameters on presentation and 72 hours after drainage g/dL: grams per deciliter, IU/L: international units per liter, mEq/L: milliequivalents per liter

Blood Parameter	On admission	At 72 hours after drainage	Reference Range
Hemoglobin	7.5 g/dL	8.6 g/dL	13.2-17.3 g/dL
Total leukocyte count	47.09 x 10³/µL	12.1 x 10³/µL	4.0-11.0 x 10³/µL
Platelet count	686 x 10³/µL	498 x 10³/µL	150-450 x 10³/µL
Serum sodium	126 mEq/L	134 mEq/L	136-146 mEq/L
Serum calcium	7.23 mg/dL	8.15 mg/dL	8.8-10.6 mg/dL
Serum amylase	5820 IU/L	1680 IU/L	28-100 IU/L
Serum lipase	6485 IU/L	2140 IU/L	0-67 IU/L

**Figure 1:**
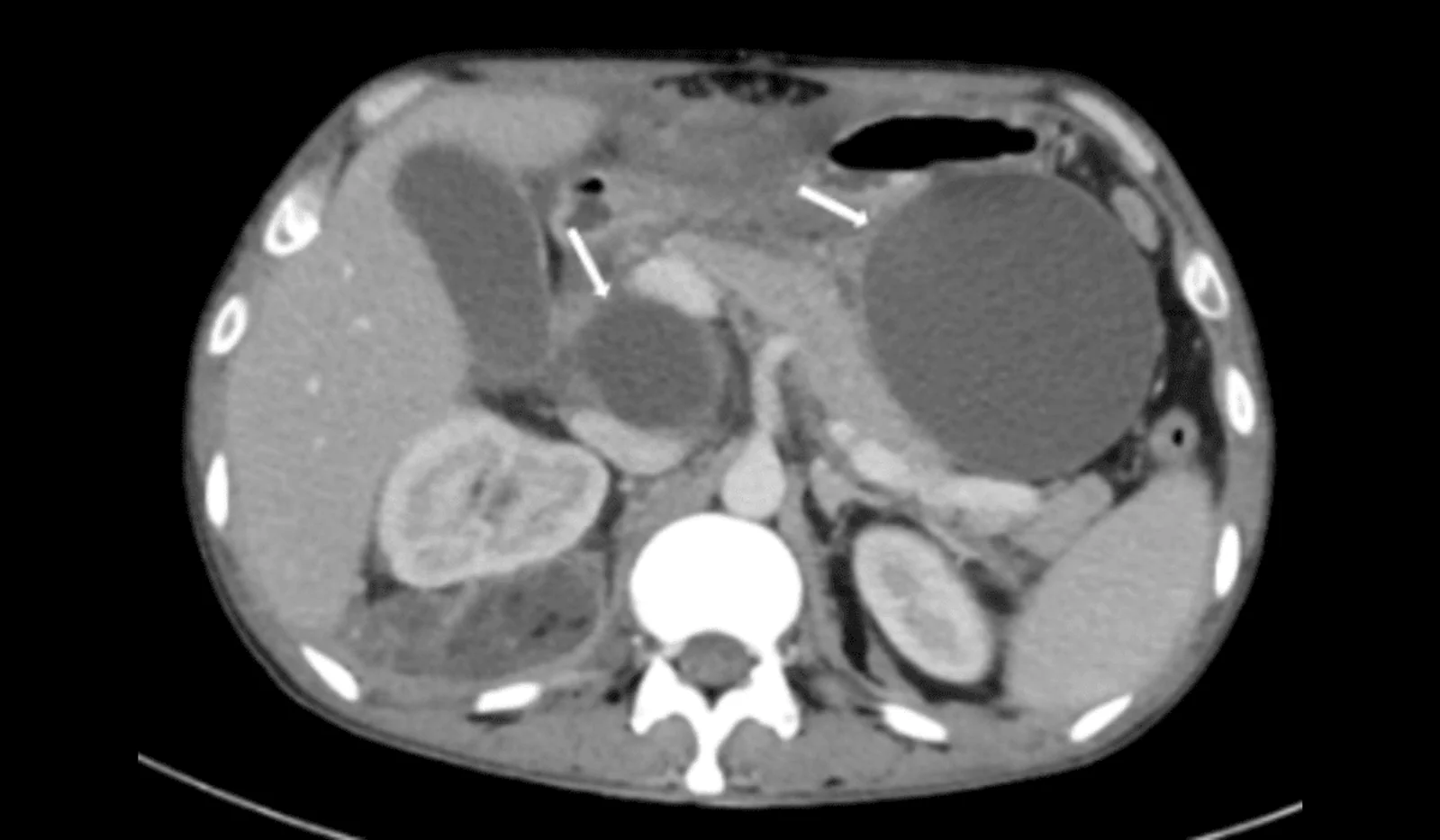
Axial section of contrast-enhanced CT of the abdomen shows a few, well-defined, peripherally enhancing collections adjacent to the pancreatic head and tail, suggestive of pancreatic pseudocysts (white arrows)

Initial imaging with contrast-enhanced CT of the thorax and abdomen, along with corroborative transabdominal ultrasound, demonstrated features of acute pancreatitis with multiple well-defined peripherally enhancing (on CT)/hypoechoic (on ultrasound) collections involving the pancreatic head and tail (Figure 1). 

**Figure 2:**
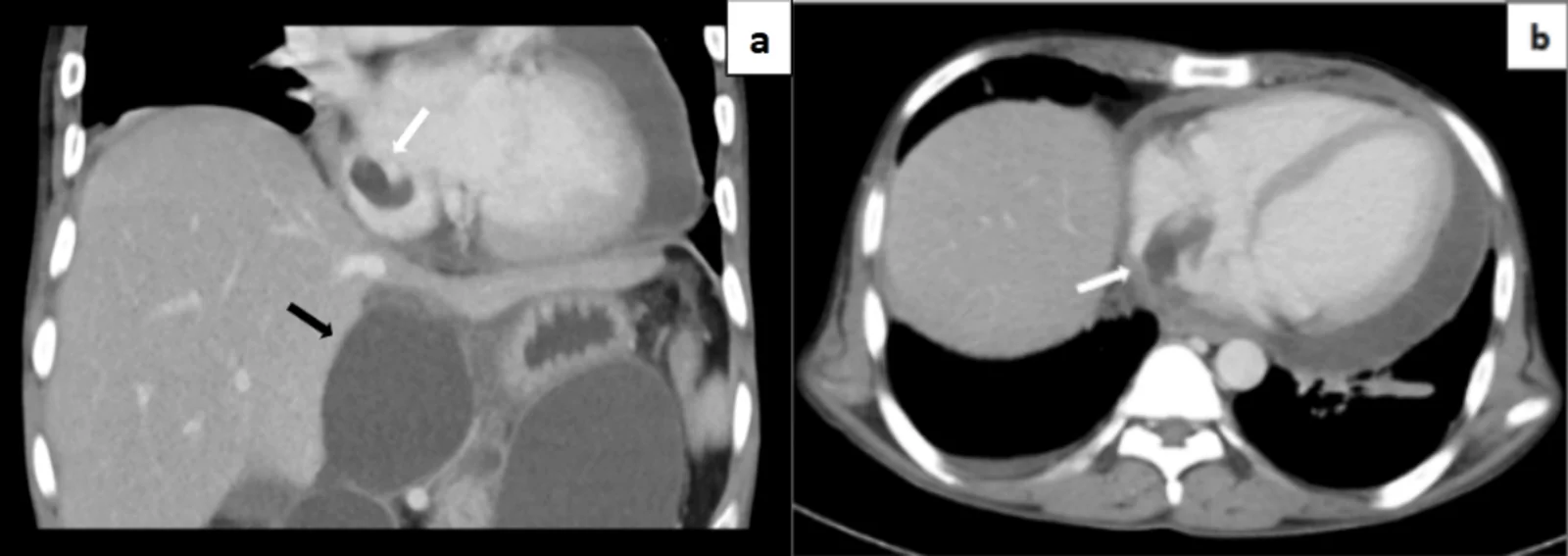
Contrast-enhanced CT of thorax and abdomen (a) Coronal section shows a large, well-defined pseudocyst (black arrow) in the abdomen and a hypodense fluid-attenuating filling defect in the right atrium (white arrow); (b) Axial sections show an irregular hypodense fluid-attenuating filling defect within the right atrial cavity (white arrow)

In addition, CT revealed a hypodense filling defect within the right atrium, which was initially interpreted as thrombus (Figure 2).

**Figure 3:**
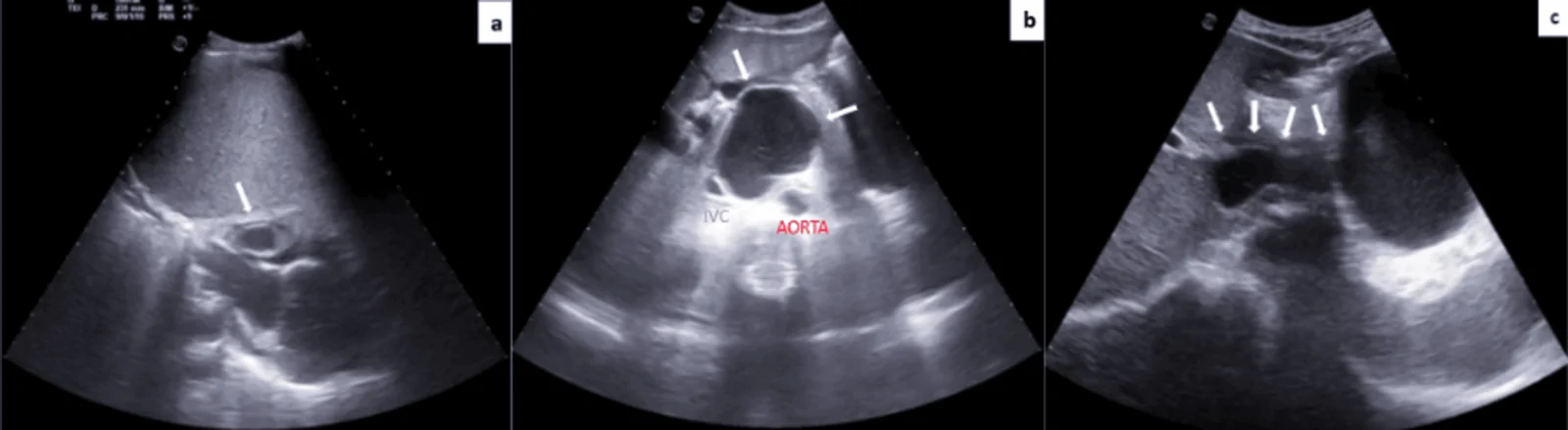
Focused ultrasound (abdominal and subxiphoid windows) (a) Thin-walled cystic structure (white arrow) within the right atrial cavity, subxiphoid four-chamber view; (b) Large abdominal collection (white arrows) in the interaortocaval space, adjacent to the hepatic inferior vena cava, subcostal transverse view; (c) The same collection extending superiorly along the course of the inferior vena cava, subcostal longitudinal view

Subsequently, a targeted ultrasound evaluation was performed for further characterization. Focused ultrasound (abdominal and subxiphoid windows) demonstrated a well-defined anechoic cystic lesion measuring approximately 22 × 11 mm within the right atrial cavity, without internal vascularity. The lesion was seen to extend inferiorly along the course of the inferior vena cava, demonstrating continuity with the abdominal pseudocyst (Figure 3).

**Figure 4:**
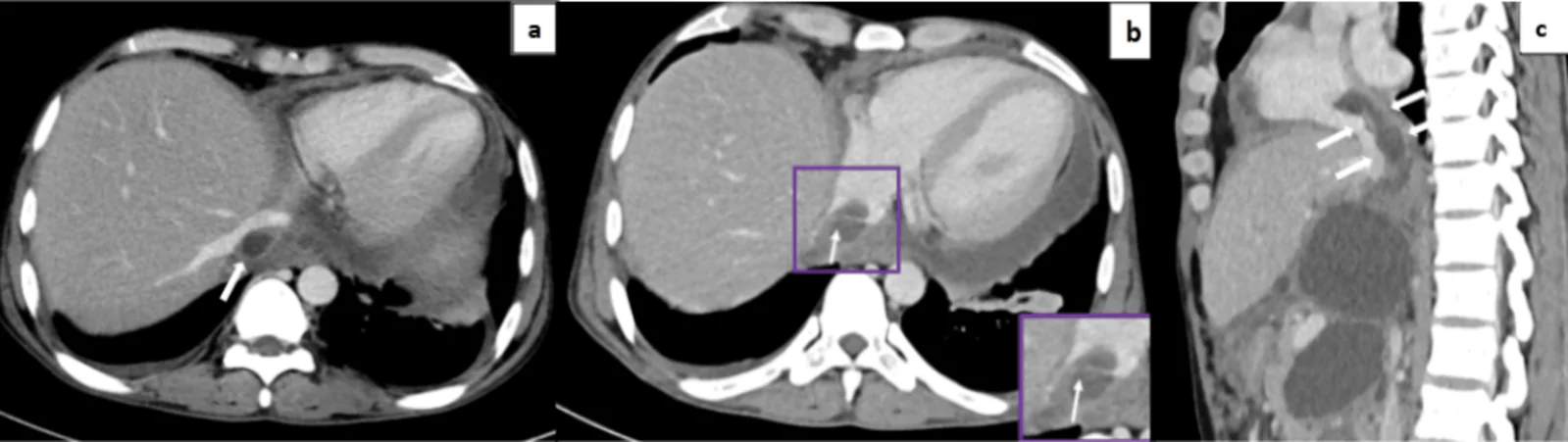
Contrast-enhanced CT of the thorax and abdomen (a) Axial section showing the collection (white arrow) adjacent to the inferior vena cava; (b) Axial section at the caval-atrial junction showing the focal full-thickness caval wall defect (white arrow) through which the collection herniates into the lumen, with its fibrous capsule bridging the defect and separating the contents from the opacified blood pool; (c) Sagittal reformat showing longitudinal extension of the collection along the periadventitial plane of the inferior vena cava and its intracavitary component within the right atrium (white arrows)

On retrospective review of the CT images following targeted ultrasound, the collection was contiguous with a fluid-attenuating structure extending cranially along the periadventitial plane of the retrohepatic inferior vena cava and through the caval hiatus. At the caval-atrial junction, the caval wall was breached, and the collection herniated through the defect into the lumen, enclosed by its own fibrous capsule, so that the intracavitary component projected into the right atrium without communication between the cyst cavity and the circulating blood. A discrete wall was demonstrable, separating the contents from the opacified blood pool. The lesion measured 15 HU, distinct from the attenuation expected of thrombus (Figure 4). No contrast entered the collection on the portal-venous phase, and the inferior vena cava showed no thrombosis, stenosis, or collateral formation.

Based on these findings, the right atrial lesion was reinterpreted as intracardiac extension of a pancreatic pseudocyst rather than thrombus, thereby avoiding inappropriate anticoagulation.

The collection extended from the interaortocaval space laterally into the left paracolic gutter, where it lay superficially beneath the abdominal wall and afforded the most direct sonographic window. Ultrasound-guided percutaneous drainage was performed via a left paracolic gutter approach using a 14 Fr pigtail catheter. Although a transmural corridor was not anatomically precluded, the percutaneous route was preferred because a transgastric tract or stent into a collection communicating with a full-thickness caval defect would risk establishing a communication between the gastrointestinal and caval lumina; percutaneous drainage additionally allowed local anesthesia alone in a patient with a marked systemic inflammatory response, and permitted external quantification of output.

**Figure 5:**
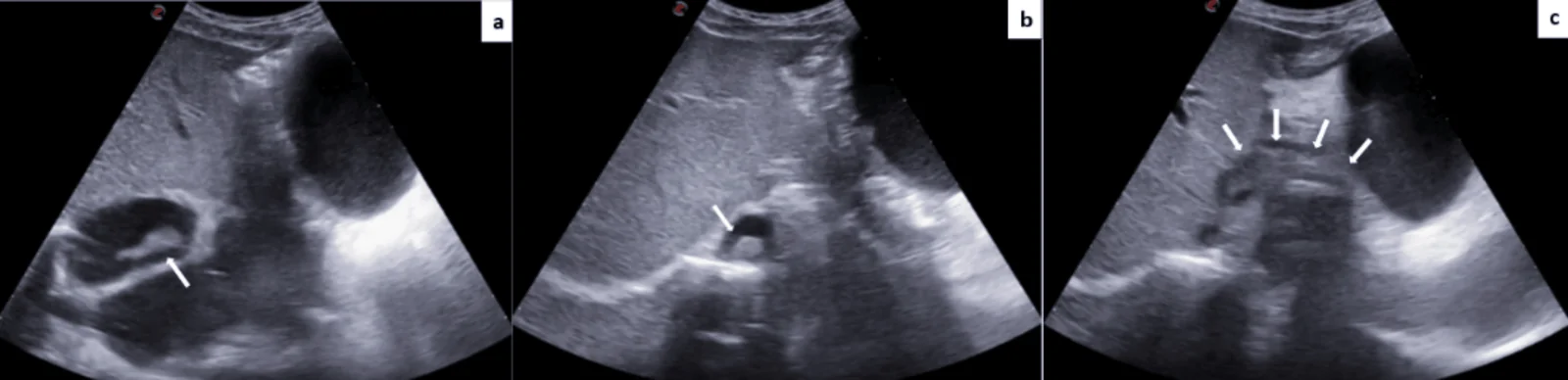
Focused ultrasound following pigtail placement and drainage (a) Linear echogenic structure (white arrow) within the right atrial cavity, representing the apposed walls of the decompressed collection, subxiphoid four-chamber view; (b) rounded echogenic structure (white arrow) within the inferior vena caval lumen, transverse subcostal view; (c) the same structure in longitudinal section (white arrows), demonstrating continuity with the abdominal component and absence of fragmentation

This yielded approximately 300 mL/day of serous, non-hemorrhagic fluid. Analysis revealed amylase 18,500 IU/L and lipase 32,500 IU/L, confirming pancreatic origin. Output fell to 40 mL/day by day 5, remaining non-hemorrhagic throughout, and the catheter was removed on day 7. Follow-up ultrasound at one week demonstrated marked reduction of the abdominal collection and near-complete collapse of the intracavitary component in continuity with it (Figure 5). The patient was discharged in stable condition and remained asymptomatic at the three-month follow-up.

## Discussion

Pancreatic pseudocysts are encapsulated collections that may extend beyond the peripancreatic region through enzymatic digestion of surrounding tissue and raised intracystic pressure, dissecting along fascial planes into adjacent compartments such as the mediastinum [3,6] and other atypical locations [4]. Extension to the caval lumen and cardiac chambers; however, remains exceptionally uncommon, and understanding it requires a distinction not usually drawn: a breach of the vessel wall is not equivalent to a communication between the vessel lumen and the cyst cavity, since the collection may carry its own wall through the defect.

An enclosed collection can still digest an adjacent vessel because a pseudocyst wall contains fluid mechanically without being a barrier to the enzymes it holds. The wall is granulation and fibrous tissue without an epithelial lining [1,2], competent to retain fluid under pressure yet permeable to the proteolytic enzymes within. The enzyme therefore degrades the collagen- and elastin-rich adventitia and media, which lie in a stagnant abluminal compartment, and ultimately the intima, producing a focal full-thickness mural defect--the process that in previously reported cases has established communication between a pseudocyst and the inferior vena cava [5,7]. We propose that erosion then arrests the enzyme from reaching flowing blood, being diluted and neutralized in the circulation, and that because intracystic pressure exceeds central venous pressure, the collection herniates inward through the defect rather than blood decompressing outward into it. On this interpretation, the pseudocyst capsule, rather than the caval wall, becomes the membrane separating cyst contents from circulating blood--an inference from the imaging appearances and clinical course rather than a directly demonstrated finding.

Three configurations are possible, and they are not equivalent. The collection may remain external and invaginate the caval or atrial wall, producing an apparent filling defect with no true intracavitary component; it may herniate into the lumen while retaining its capsule, as here; or free fistulous communication may be established, which would be expected to manifest with systemic enzyme effects, consumptive coagulopathy, caval thrombosis, and sepsis. Reported cases of pseudocyst-caval fistula illustrate this third pattern, with caval stenosis, renal vein thrombosis, and dysfibrinogenemia [7], and frank rupture into the caval lumen [5]. Our patient exhibited none of these: coagulation remained normal, the cava was patent and undistorted, there was no clinical sepsis, and the aspirate was serous and non-hemorrhagic. The clinical course is decisive: decompression of the abdominal component alone produced collapse of the intracavitary component, behavior possible only if the two are hydraulically continuous within a single-walled cavity, and one that neither thrombus, an extrinsic collection, nor a free communication would show.

The differential for a right atrial filling defect includes thrombus, myxoma, infective vegetation, and metastasis. Myxoma, vegetation, and metastasis are vascularized or echogenic solid lesions and were excluded by the anechoic, avascular character of the lesion and its continuity with an abdominal collection. Thrombus, the initial interpretation, was excluded by the fluid attenuation of the lesion at 15 HU and by its continuity with a collection whose aspirate contained amylase 18,500 IU/L and lipase 32,500 IU/L, confirming pancreatic origin, and by its subsequent regression after drainage of the abdominal collection alone. The case illustrates the complementary roles of the two modalities, ultrasound affording real-time assessment of cystic morphology and vascularity and CT delineating anatomical extent and relationships [2].

Recognition carries therapeutic weight: misdiagnosis invites inappropriate anticoagulation or unnecessary surgery, whereas percutaneous drainage here produced regression of both components and confirmed the diagnosis. Management depends on symptoms, size and complications. Conservative management suits small asymptomatic collections but is inappropriate where an intracavitary component is present, given the potential for progressive extension and diagnostic uncertainty; endoscopic transmural drainage is preferred for mature collections apposed to the stomach or duodenum; and surgical intervention, though reported for intrathoracic and intracardiac extension, carries greater morbidity and may be avoided entirely when the intracavitary component is a contained extension of a drainable abdominal collection. Minimally invasive drainage therefore remains the cornerstone in appropriately selected patients [4].

Two cautions merit statement. First, embolization of collapsed cyst wall material remains a theoretical hazard of this configuration; although no fragmentation or migration occurred in our patient, this argues for close post-procedural surveillance with a low threshold for cross-sectional reassessment, and an uncomplicated course in a single patient should not be read as evidence of general safety. Second, the distinction between contained herniation and free communication should be actively sought before intervention, since the latter would materially alter the risk profile of percutaneous drainage [5,7]; useful discriminators would include absence of contrast within the collection on delayed-phase imaging, absence of blood within the aspirate, and a patent, undistorted cava.

This report has certain limitations. The mechanism is inferred from imaging and clinical course; no operative, angiographic, or histopathological confirmation was obtained, and deep extrinsic invagination cannot be excluded with certainty, although it is difficult to reconcile with the persistence of a collapsed intracavitary structure after remote decompression. The composition of the membrane separating cyst contents from blood, whether capsule, residual intima, or both in apposition, lies below the resolution of the modalities used.

## Conclusions

Pancreatic collections may rarely extend along the inferior vena cava into the right atrium, where they closely mimic intracardiac thrombus. Enzymatic erosion may breach the caval wall in full thickness while the collection herniates into the lumen enclosed by its own fibrous capsule, so that a mural breach exists without communication between the cyst cavity and the circulating blood. Recognizing this configuration, and distinguishing it from both extrinsic invagination and true fistulous communication, governs the complications to be anticipated and the safety of intervention. The diagnosis rests on demonstrating continuity with an abdominal collection, fluid attenuation, and absence of internal vascularity. Where the intracavitary component is a contained extension of a drainable abdominal collection, image-guided percutaneous decompression may resolve both, avoiding inappropriate anticoagulation and unnecessary surgical intervention.
